# Risk Indicators Affecting the Survival of the Mandibular First Molar Adjacent to an Implant at the Mandibular Second Molar Site: A Retrospective Study

**DOI:** 10.3390/jcm10122543

**Published:** 2021-06-08

**Authors:** Won-Bae Park, Koo-Hyun Kwon, Kyung-Gyun Hwang, Ji-Young Han

**Affiliations:** 1 Department of Periodontology, School of Dentistry, Kyung Hee University, Seoul 02447, Korea; njkysh@naver.com; 2 Private Practice in Periodontics and Implant Dentistry, Seoul 02787, Korea; 3 Department of Dentistry, Division of Prosthodontics, Hanyang University Medical Center, Seoul 04763, Korea; sartna@naver.com; 4 Department of Dentistry, Division of Oral & Maxillofacial Surgery, College of Medicine, Hanyang University, Seoul 04763, Korea; hkg@hanyang.ac.kr; 5 Department of Dentistry, Division of Periodontology, College of Medicine, Hanyang University, Seoul 04763, Korea

**Keywords:** dental occlusion, dental implant, furcation defects, periodontal disease, survival, tooth wear

## Abstract

This study aimed to compare the survival of mandibular first molars (MnM1s) adjacent to implants placed in mandibular second molar sites (ImM2s) with MnM1s adjacent to mandibular second molars (MnM2s) and to investigate risk indicators affecting the survival of MnM1s adjacent to ImM2s. A total of 144 patients who had MnM1s adjacent to ImM2s and MnM1s adjacent to MnM2s on the contralateral side were included in this study. Clinical variables and radiographic bone levels were evaluated. The survival of MnM1s adjacent to ImM2s or MnM2s was evaluated using a Kaplan–Meier analysis and Cox proportional hazards model. The 5-year cumulative survival rates of MnM1s adjacent to ImM2s and MnM2s were 85% and 95%, respectively. MnM1s adjacent to ImM2s of the internal implant-abutment connection type had higher multivariate hazard ratios (HR) for loss. MnM1s that had antagonists with implant-supported prostheses also had higher HR for loss. The multivariate HR for the loss of MnM1s adjacent to ImM2s with peri-implant mucositis was 3.74 times higher than MnM1s adjacent to healthy ImM2s. This study demonstrated several risk indicators affecting the survival of MnM1s adjacent to ImM2s. It is suggested that supportive periodontal and peri-implant therapy combined with meticulous occlusal adjustment can prolong the survival of MnM1s and ImM2s.

## 1. Introduction

Single implant treatment is considered a predictable restorative treatment option [1,2,3]. When a posterior single tooth is missing, single implants are the most biomimetic design.

After the delivery of an implant-supported prosthesis, however, biological and mechanical complications associated with the implant are frequently encountered [4]. Dental/implant occlusion is not a simple contact between opposite surfaces, and includes the friction of multiple inclined planes and different force vectors [5]. Dental implants that do not have periodontal ligament or periodontal mechanoreceptors are more susceptible to bending loads compared to the natural teeth [6,7]. When the occlusal load applied through function or parafunctions exceeds the resistance of the prosthesis, implant components, implant and osseointegrated interface, occlusal overload occurs, causing structural or biological damage [8]. In addition, it is reported that dental implants are thought to be more prone to occlusal overloading than natural teeth because of the loss of the periodontal ligament, which provides shock absorption and periodontal mechanoreceptors, providing tactile sensitivity and proprioceptive motion feedback [9]. Therefore, the occlusal concept applied to the implant-supported prosthesis needs to be different from that of natural teeth because implants react biomechanically in a different fashion to occlusal force [10].

Natural teeth continue to erupt throughout their lives [11,12,13,14]. In contrast, because of the direct contact between the implant and bone, implants do not follow these tooth movements. The continuous eruption and physiological movement of tooth adjacent implants may result in minor occlusal change. Therefore, it may result in traumatic occlusion or overloading in the teeth adjacent implants [15,16,17]. However, the wear on the adjacent teeth and the opposing teeth may compensate for the continuous tooth eruption. The human dentition is a dynamic system being continuously exposed to masticatory and interdental forces [18]. In addition, it is considered that continuous eruption might have evolved as a compensatory mechanism for heavy occlusal wear [18]. The wear of tooth and restoration is influenced by various factors including biological, mechanical, and chemical components [19,20,21]. Therefore, the degree of wear depending on the type of prosthetic material may influence the occlusal change of the tooth adjacent to an implant.

However, to the best of our knowledge, there have been few studies about the survival of the teeth adjacent to the most posterior mandibular implants. Therefore, the purpose of this study was to compare the survival of the mandibular first molars adjacent to implants placed in the mandibular second molar sites with the mandibular first molars adjacent to the mandibular second molars, and to investigate the risk indicators affecting the survival of the mandibular first molars adjacent to implants placed in the mandibular second molar sites.

## 2. Materials and Methods

### 2.1. Subjects

**Figure 1:**
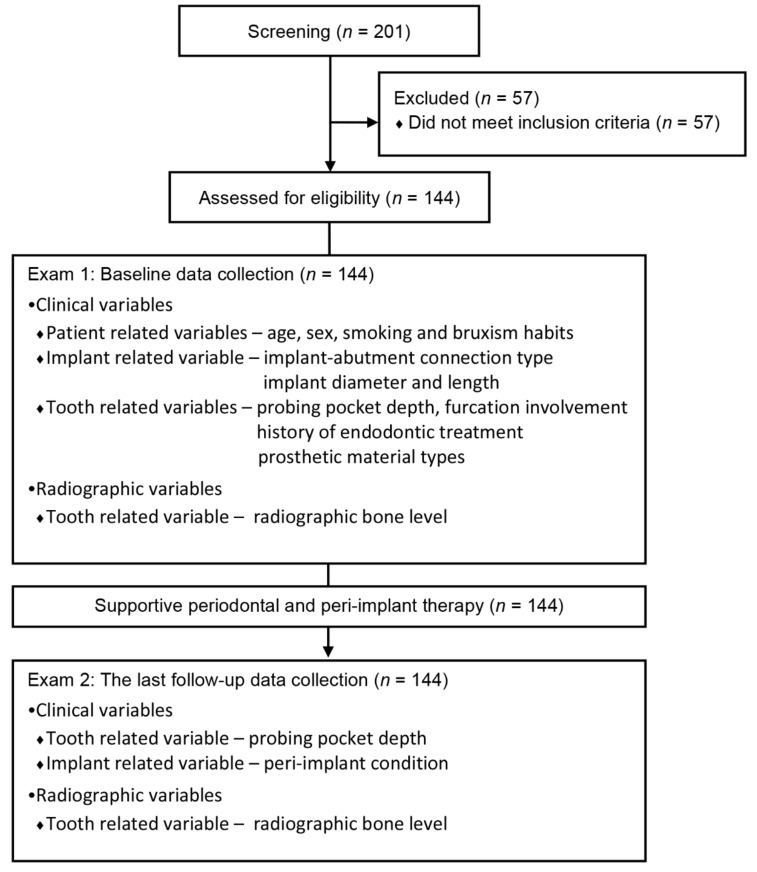
Flow chart of the study.

All patients who underwent single implant placement at the private practice and the Department of Periodontology of Hanyang University Medical Center between March 2000 and August 2019 were screened for eligibility for this study (Figure 1).

Inclusion criteria:

- Patients who were more than 20 years old
- Patients who had no relevant systemic conditions or diseases
- Patients who had the mandibular first molars (MnM1s) adjacent to the single implants placed in the mandibular second molar sites (ImM2s)
- Patients who had the mandibular first molars (MnM1s) and the mandibular second molars (MnM2s) on the contralateral side
- Patients who had been followed up at least 2 years after prosthesis delivery of ImM2s
- Patients who had periapical radiographs after prosthesis delivery and before extraction of MnM1s or the last follow-up visits

Exclusion criteria:

- Patients who had an active infection or disease affecting bone metabolism and wound healing
- Patients who had regular use of steroids or other medications affecting bone turnover

This study was approved by the Institutional Review Board of Hanyang University Hospital (HYUH-2020-04-058). STROBE (Strengthening the Reporting of Observational Studies in Epidemiology) guidelines were observed for the preparation of the manuscript.

### 2.2. Clinical Variables

The following variables were evaluated using patient clinical records. Patient-related variables including age, sex, smoking and bruxism habit were assessed. Patients were considered as presenting “possible” sleep or awake bruxism based on self-reporting using questionnaires and/or the anamnestic part of a clinical examination [22].

Implant-related variables including the implant diameter, implant length, implant-abutment connection type (external vs. internal type) and peri-implant condition of the ImM2 were recorded. The peri-implant condition of ImM2 was classified as healthy, peri-implant mucositis and peri-implantitis [23].

The periodontal status of MnM1 including probing pocket depth (PPD), degree of furcation involvement [24] and endodontic status of MnM1 were assessed using clinical records. Prosthetic material types of the MnM1 (natural tooth, gold crown or porcelain fused metal crown) and the antagonist (natural tooth, gold crown, porcelain fused metal crown or implant-supported prosthesis with porcelain fused metal crown) of the MnM1 adjacent to the ImM2 or the MnM2 were evaluated.

### 2.3. Radiographic Measurements

**Figure 2:**
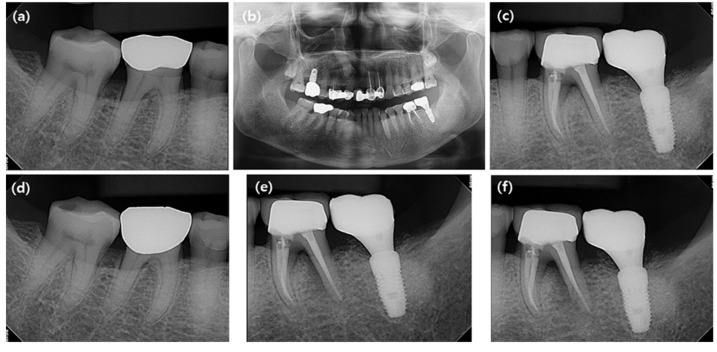
Radiographic measurement. (**a**) A periapical radiograph of the mandibular first molar (MnM1) adjacent the mandibular second molar (MnM2) at baseline. (**b**) A panoramic radiograph at baseline. (**c**) A periapical radiograph of MnM1 adjacent to the single implant placed in the mandibular second molar site (ImM2) at baseline. (**d**) A periapical radiograph of MnM1 adjacent MnM2 at the last follow-up. (**e**) A periapical radiograph of MnM1 adjacent to ImM2 at 3-year follow-up after prosthesis delivery of ImM2. (**f**) A periapical radiograph of MnM1 adjacent to ImM2 at the last follow-up.

The radiographic evaluation was performed using a digital radiography system (CS9300 Select, Carestream Dental LLC, Atlanta, GA, USA). Panoramic and periapical radiographs taken immediately after prostheses delivery of ImM2s (baseline) and at the last follow-up visits (or immediately before the extraction of MnM1s adjacent to ImM2s or MnM2s) were used for radiographic assessment (Figure 2).

Panoramic and periapical radiographs were imported into Analysis Toolkit (Adobe Photoshop CS6, Adobe Systems Inc., San Jose, CA, USA). Radiographic bone levels of the MnM1s adjacent to ImM2s or MnM2s were assessed at the mesial and distal sites using the baseline and the last follow-up (or immediately before the extraction of MnM1s adjacent to ImM2s or MnM2s) periapical radiographs. One blinded examiner (K.-G. H.) twice measured the distance between the cemento-enamel junction and the most coronal level of the alveolar bone of MnM1s for all sets of periapical radiographs at an interval of at least two weeks. The mean of the two measurements was used as the radiographic bone level value. Intra-class correlation coefficients (ICCs) were used to test the reproducibility of the radiographic measurements.

### 2.4. Survival of Tooth Adjacent to Single Implant

The survival of the MnM1 adjacent to the ImM2 or the MnM2 was evaluated. The follow-up period until the MnM1 adjacent to the ImM2 or the MnM2 had been extracted was recorded. The interval time from the date of the prosthesis delivery of ImM2 to the date of extraction of the MnM1 adjacent to the ImM2 or the MnM2 was assessed in months.

### 2.5. Statistical Analysis

All statistical analyses were performed using SPSS (version 21, IBM Corp., Armonk, NY, USA). Descriptive statistics were performed using means ± standard deviations (SDs) or medians (first quartile, third quartile) for quantitative variables. After normality testing using the Shapiro–Wilk test, the differences in the clinical parameters of MnM1s between baseline and the last follow-up visit were analyzed using the Wilcoxon signed rank test. Differences in probing pocket depths and radiographic bone levels of MnM1s according to the implant-abutment connection type and endodontic treatment status were analyzed using the Mann–Whitney U test. The Kruskal–Wallis test was used to analyze the differences in probing pocket depth and radiographic bone level of MnM1 according to the degree of furcation involvement, endodontic treatment status, the prosthetic material types of MnM1 and the antagonist of MnM1. The cumulative survival rate of MnM1s adjacent to ImM2s or MnM2s was estimated using a Kaplan–Meier analysis. The log-rank test was used to identify the significant differences in the survival functions between the groups. Univariate and multivariate Cox proportional hazards regression models were used to analyze the hazard ratios for the loss of MnM1s adjacent to ImM2s or MnM2s. A *p*-value less than 0.05 was considered statistically significant.

## 3. Results

A total of 144 patients (89 men and 55 women) with a mean age of 54.49 ± 9.59 years (range: 27 to 75 years) were included in the present study (Table 1).

Six patients were lost to follow-up. Thirty-eight patients (26.39%) were smokers, and 34 patients (23.61%) had bruxism habits. The mean follow-up period was 6.94 ± 3.76 years (range: 2.75 to 19.67 years). The ICC for the radiographic measurement of the bone level was 0.938. Forty-one MnM1s adjacent to ImM2s were extracted and 16 MnM1s adjacent to MnM2 were extracted (Table 2). The reasons for extraction of MnM1s adjacent to ImM2 were endodontic origin (n = 6), periodontal origin with or without endodontic origin (n = 31) and tooth fracture (n = 4).

**Figure 3:**
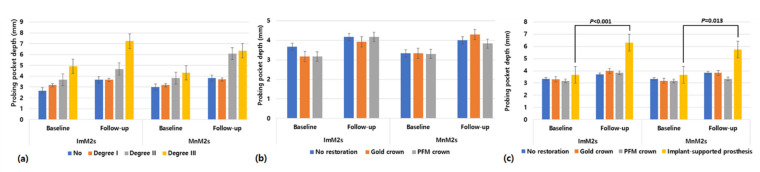
Changes in probing pocket depths of the mandibular first molars (MnM1s) between baseline and the last follow-up according to (**a**) furcation involvement of MnM1s, (**b**) prosthetic type of MnM1s, and (**c**) prosthetic type of antagonists. Abbreviations: ImM2s, implants placed in the mandibular second molar site; MnM2s, the mandibular second molars; PFM, porcelain fused metal crown.

There were significant differences in the changes in PPD for MnM1s according to the prosthetic material types of the antagonists in both sides (ImM2s: *p* < 0.001, MnM2s: *p* = 0.013; Figure 3).

**Figure 4:**
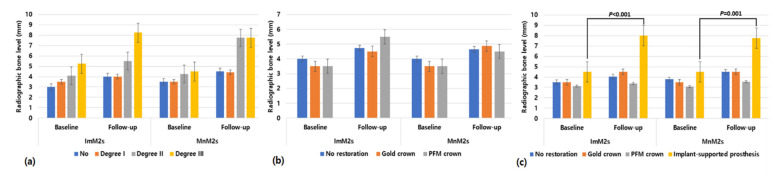
Changes in radiographic bone levels of the mandibular first molars (MnM1s) between baseline and the last follow-up according to (**a**) furcation involvement of MnM1s, (**b**) prosthetic type of MnM1s, and (**c**) prosthetic type of antagonists. Abbreviations: ImM2s, implants placed in the mandibular second molar site; MnM2s, the mandibular second molars; PFM, porcelain fused metal crown.

Regarding the radiographic bone level, MnM1s showed significantly greater radiographic bone loss when the antagonist was an implant-supported prosthesis in both sides (ImM2s: *p* < 0.001, MnM2s: *p* = 0.001; Figure 4).

**Figure 5:**
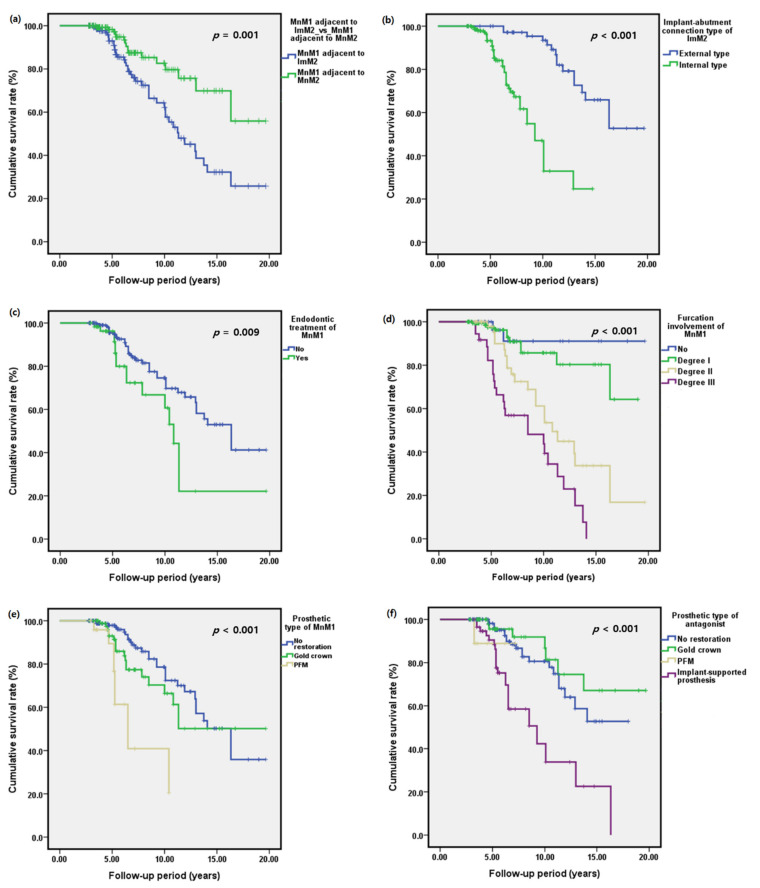
Kaplan–Meier survival curves. (**a**) The cumulative survival rate of the mandibular first molars (MnM1s) adjacent to implants (ImM2s) was significantly lower than MnM1s adjacent to the mandibular second molars (MnM2s) at the 5-year follow-up. The cumulative survival rate of the mandibular first molars adjacent to ImM2s or MnM2s according to (**b**) implant-abutment connection type of ImM2s, (**c**) history of endodontic treatment of MnM1s, (**d**) degree of furcation involvement of MnM1s, (**e**) the prosthetic material types of MnM1s and (**f**) the prosthetic material types of the antagonists of MnM1s.

The cumulative survival rate of MnM1s adjacent to ImM2s was 85% (95% confidence interval [CI]: 84.35–85.65%) at 5 years after the implant prosthesis delivery of ImM2s (Figure 5).

The 5-year cumulative survival rate of MnM1s adjacent to MnM2s was 95% (95% CI: 94.67–95.33%). The median survival time of MnM1s adjacent to ImM2s (11.33 years) was significantly shorter than MnM1s adjacent to MnM2s (*p* = 0.001; log-rank test; Figure 5). The median survival time of MnM1s adjacent to MnM2s or ImM2s after the implant prosthesis delivery of ImM2s with an internal implant-abutment connection type (9.25 years) was significantly shorter than MnM1s adjacent to ImM2s with an external implant-abutment connection type (*p* < 0.001; log-rank test; Figure 5). The median survival time of MnM1s with furcation involvement of degree III (8.5 years) was significantly shorter than those of MnM1s with no, degree I and degree II furcation involvement (*p* < 0.001; log-rank test). There were significant differences in the median survival times of MnM1s according to the prosthetic material type of the MnM1s (*p* < 0.001; log-rank test). When the antagonist of the MnM1 was an implant-supported prosthesis, the median survival time of MnM1s was significantly shorter compared to those with other prosthetic types of antagonists (*p* < 0.001; log-rank test).

Univariate and multivariate Cox regression models are shown in Table 3.

MnM1s adjacent to MnM2s showed lower multivariate hazard ratios for tooth loss (0.42; 95% CI: 0.23–0.77; *p* = 0.005) compared to MnM1s adjacent to ImM2. Patients with ImM2s of the internal implant-abutment connection type had a significantly higher multivariate hazard ratio for the loss of MnM1 (4.76; 95% CI: 1.99–11.38; *p <* 0.001) compared to patients with ImM2s of the external implant-abutment connection type. The multivariate HR for the loss of MnM1s adjacent ImM2s with peri-implant mucositis was 3.74 (95% CI: 1.42–9.89; *p* = 0.008) times higher than MnM1s adjacent to healthy ImM2s. The MnM1s with degree III furcation involvement showed a significantly higher multivariate hazard ratio for tooth loss (13.23; 95% CI: 2.71–64.59; *p* = 0.001). MnM1s restored with PFM had a higher multivariate hazard ratio for tooth loss (6.66; 95% CI: 2.17–20.45; *p* = 0.001). Patients with an antagonist of implant-supported prostheses with ceramo-metal had a higher multivariate hazard ratio for the loss of MnM1 adjacent to ImM2 (5.62; 95% CI: 2.72–11.63; *p <* 0.001).

## 4. Discussion

The results of this study showed that MnM1s adjacent to ImM2s exhibited higher hazard ratios for tooth loss compared to MnM1s adjacent to MnM2s. In addition, MnM1s adjacent to ImM2s or MnM2s exhibited higher hazard ratios for tooth loss when the MnM1s had a PFM crown, an antagonist with implant-supported prosthesis, an ImM2 of the internal implant-abutment connection type or an inflammatory peri-implant condition.

Dental implants do not have periodontal ligaments and are osseointegrated by direct bone-to-implant contact, similar to an ankylosed tooth [25,26]. On the other hand, teeth continue to erupt throughout life [11,12,13,14]. The continuous eruption and physiological movement of tooth adjacent implants may result in minor occlusal change. Tooth wear caused by multiple factors including biological, mechanical and chemical components [21] may compensate for the continuous tooth eruption. Wear patterns vary according to occlusion, joint pathology, muscle tone, lubricants, individual dietary habits and prosthetic material type [27,28,29]. Wear properties of restorative dental materials are reported to be different from those of natural teeth [30,31,32]. Additionally, tooth wear can be influenced by the wear rate of antagonists.

In this study, we evaluated the prosthetic material types of MnM1s and antagonist of MnM1s. The MnM1s restored with PFM crowns had a higher multivariate hazard ratio for tooth loss (6.66; 95% CI: 2.17–20.45; *p* = 0.001) than other restorative materials. Hacker et al. [33] reported that conventional feldspathic porcelain (230 ± 38 μm) caused enamel to wear more than gold did (9 ± 13 μm). The MnM1s had a significantly higher multivariate hazard ratio for tooth loss (5.62; 95% CI: 2.72–11.63; *p* < 0.001) when antagonists were implant-supported prostheses compared to natural teeth. It has been reported that wear will not occur unless occlusal stress exceeds the strength of the opposing materials, because wear requires the sliding of one surface against the other [19].

The present study showed that the MnM1s with ImM2s of the internal implant-abutment connection type had a significantly higher multivariate hazard ratio for tooth loss (4.76; 95% CI: 1.99–11.38; *p <* 0.001) compared to the MnM1s with ImM2s of the external implant-abutment connection type. Lee et al. [34] reported that the internal tapered conical connection demonstrated a variable amount of axial displacement with tightening torque and cyclic loading. Furthermore, Ko et al. [35] suggested that the long-term axial displacement of internal conical connection implants should be carefully managed.

Natural teeth have periodontal mechanoreceptors that signal information used for the fine motor control of jaw actions associated with biting, intraoral manipulation and the chewing of food [36]. However, important sensory-motor functions are lost or impaired when periodontal ligament receptors are reduced or eliminated through periodontal breakdown, bruxism, chewing, extraction and anesthesia [36,37]. Thus, a patient’s ability to detect occlusal inaccuracies induced by restorative treatment may be decreased, and inappropriate exteroceptive feedback may thus present a risk for overloading the prosthesis [37]. After the placement of dental implants, it has been reported that the detection thresholds of implant prostheses are increased to a thickness of at least 50–100 µm and 50–100 g upon tooth loading [37]. Thus, several contributing factors including the wear of restoration and the loss of tactile function of an implant may influence the occlusal trauma of an MnM1 adjacent to an ImM2.

An observational study showed that traumatic occlusal forces may be associated with the severity of periodontitis [38]. Additionally, Branschofsky et al. [39] reported that secondary trauma from occlusion is frequently seen in periodontally compromised patients and is positively correlated with the severity of attachment loss. Although a recent consensus reported that there is no evidence that traumatic occlusal forces can accelerate the progression of periodontitis in humans [40], it was reported that occlusal trauma can activate IKK-NF-κB signaling, which may result in the inhibition of osteogenic differentiation in vitro and bone formation in vivo [41].

The MnM1s with grade III furcation involvement showed a significantly greater multivariate hazard ratio for tooth loss (13.23; 95% CI: 2.71–64.59; *p* = 0.001). This result was similar to a previous study which showed that molars with furcation involvement had an increased risk of tooth loss during supportive periodontal therapy (OR 5.26; 95% CI: 1.46–19.03; *p* = 0.012) [42]. In addition, we found that the multivariate HR for the loss of MnM1s adjacent to ImM2s with peri-implant mucositis was 3.74 (95% CI: 1.42–9.89; *p* = 0.008) times higher than MnM1s adjacent to healthy ImM2s.

In the current study, we aimed to investigate the influence of bruxism on occlusal trauma. The univariate hazard ratio for the loss of MnM1s with a bruxism habit was 1.8 (95% CI: 1.02–3.19; *p* = 0.043). However, we failed to find any significant differences in multivariate hazard ratios for the loss of MnM1s between non-bruxism and bruxism patients.

This study had several strengths including a long-term follow-up period, univariate and multivariate evaluations of risk indicators to minimize confounding factors and tribological evaluation of prostheses. The limitation of the present study was the lack of histological confirmation of occlusal trauma, which is an inherent bias of retrospective studies. In addition, bruxism habits were evaluated by patients’ self-reporting using questionnaires and/or the anamnestic part of a clinical examination without a polysomnographic or an electromyographic evaluation. Therefore, the findings of this study should be interpreted with caution. Future prospective studies with large sample sizes are recommended. Nonetheless, the results of this study can be used as a basis for understanding the occlusal trauma of the MnM1s adjacent to ImM2s and the possible risk indicators for the survival of MnM1s adjacent to ImM2s.

## 5. Conclusions

This study demonstrated that MnM1s adjacent to ImM2s exhibited higher hazard ratios for tooth loss compared to MnM1s adjacent to MnM2s and identified several risk indicators affecting the survival of MnM1s adjacent to ImM2s. These risk indicators were the internal type of implant-abutment connection, PFM crown restoration of MnM1, antagonists with implant-supported prostheses and inflammatory peri-implant conditions. Therefore, it is suggested that supportive periodontal and peri-implant therapy combined with continuous and meticulous occlusal adjustment can prolong the survival of an implant and the adjacent tooth.

## Figures and Tables

**Table 1:** Patient demographic characteristics.

Variables		N	
Age (years)	<50 years	44 (30.56%)	
	≥50 years	100 (69.44%)	
	(Mean ± SD)	(54.49 ± 9.59)	
Sex	Male	89 (61.81%)	
	Female	55 (38.19%)	
Smoking	Non-smoker	106 (73.61%)	
	Smoker	38 (26.39%)	
Bruxism	No	110 (76.39%)	
	Yes	34 (23.61%)	
Implant-abutment connection type	External type	39 (27.08%)	
	Internal type	105 (72.92%)	
Peri-implant condition of ImM2	Healthy	68 (47.22%)	
	Peri-implant mucositis	65 (45.14%)	
	Peri-implantitis	11 (7.64%)	
Distance between ImM2 and MnM1	<3 mm	0 (0%)	
	≥3 mm	144 (100%)	
Follow-up (years)	(Mean ± SD, range)	(6.94 ± 3.76, 2.75–19.67)	
		MnM1 adjacent to ImM2	MnM1 adjacent to MnM2
Endodontic treatment	No	114 (79.17%)	118 (81.94%)
	Yes	30 (20.83%)	26 (18.06%)
Furcation involvement	No	15 (10.42%)	19 (13.19%)
	Degree I	74 (51.39%)	85 (59.03%)
	Degree II	33 (22.92%)	26 (18.06%)
	Degree III	22 (15.28%)	14 (9.72%)
Prosthetic type	no restoration	84 (58.33%)	87 (60.42%)
	gold crown	47 (32.64%)	46 (31.94%)
	PFM	13 (9.03%)	11 (7.64%)
Prosthetic type of antagonist	no restoration	77 (53.47%)	79 (54.86%)
	gold crown	35 (24.31%)	31 (21.53%)
	PFM	3 (2.08%)	6 (4.17%)
	Implant-supported prosthesis	29 (20.14%)	28 (19.44%)

Abbreviations: N, number; SD, standard deviation; MnM1s, the mandibular first molars; ImM2s, implants placed in the mandibular second molar site; MnM2s, the mandibular second molars; PFM, porcelain fused metal crown.

**Table 2:** The etiology of extraction of the mandibular first molars.

			MnM1s Adjacent to ImM2s	MnM1s Adjacent to MnM2
Extraction	No		103	128
	Yes		41	16
		Endodontic origin	6	6
		Periodontal origin (with or without endodontic origin)	31	10
		Root fracture	4	0

Abbreviations: MnM1s, the mandibular first molars; ImM2s, implants placed in the mandibular second molar site; MnM2s, the mandibular second molars.

**Table 3:** Cox proportional hazard ratio for the loss of the mandibular first molar adjacent to an implant placed in the mandibular second molar site.

Variable		Univariate HR	95% CI	*p*-Value	Multivariate HR	95% CI	*p*-Value
Age	<50 years	1			1		
	≥50 years	1.64	0.89–3.02	0.115	1.64	0.89–3.03	0.116
Sex	Male	1			1		
	Female	0.76	0.43–1.36	0.358	0.57	0.25–1.31	0.187
Smoking	No	1			1		
	Yes	1.21	0.70–2.09	0.489	1.02	0.45–2.30	0.957
Bruxism	No	1			1		
	Yes	1.8	1.02–3.19	**0.043**	1.16	0.58–2.36	0.673
Mn. 2nd molar site							
Implant supported crown		1			1		
Natural teeth		0.39	0.22–0.69	**0.001**	0.42	0.23–0.77	**0.005**
Implant-abutment connection type							
External type		1			1		
Internal type		1.49	0.84–2.66	0.173	4.76	1.99–11.38	**<0.001**
Implant health							
Healthy		1		0.419	1		**0.029**
Peri-implant mucositis		1.1	0.51–2.34	0.812	3.74	1.42–9.89	0.008
Peri-implantitis		1.5	0.74–3.06	0.262	2.62	0.98–6.98	0.054
Endodontic treatment of MnM1							
No		1			1		
Yes		2.12	1.18–3.82	**0.012**	1.4	0.50–3.93	0.518
Furcation involvement of MnM1							
0		1		**<0.001**	1		**<0.001**
Degree I		1.93	0.43–8.71	0.395	1.82	0.37–8.92	0.463
Degree II		6.52	1.52–28.01	0.012	5.23	1.11–24.63	0.036
Degree III		14.03	3.28–60.08	<0.001	13.23	2.71–64.59	0.001
Prosthetic type of MnM1							
No restoration		1		**<0.001**	1		**0.004**
Gold crown		1.45	0.81–2.59	0.207	2.79	1.18–6.61	0.019
PFM		5.42	2.49–11.79	<0.001	6.66	2.17–20.45	0.001
Antagonist prosthetic type of MnM1							
No restoration		1		**<0.001**	1		**<0.001**
Gold crown		0.64	0.27–1.51	0.306	0.87	0.32–2.31	0.771
PFM		2.39	0.32–18.08	0.397	0.24	0.02–2.62	0.242
Implant-supported prosthesis		3.39	1.93–6.00	<0.001	5.62	2.72–11.63	<0.001

Note: Bolded font, statistically significant at *p*-value <0.05. Abbreviations: HR, hazard ratio; CI, confidence interval; MnM1, the mandibular first molar; PFM, porcelain fused metal crown.

## Data Availability

The data presented in this study are available upon request from the corresponding author. The data are not publicly available because of privacy restrictions.
